# Characterization of a Newly Available Coastal Marine Dissolved Organic Matter Reference Material (TRM-0522)

**DOI:** 10.1021/acs.analchem.2c05304

**Published:** 2023-04-13

**Authors:** Stacey L. Felgate, Alexander J. Craig, Lindon W. K. Moodie, Jeffrey Hawkes

**Affiliations:** † Analytical Chemistry, Department of Chemistry BMC, Uppsala University, Uppsala 752 37, Sweden; ‡ Drug Design and Discovery, Department of Medicinal Chemistry, Uppsala University, Uppsala 752 37, Sweden

## Abstract

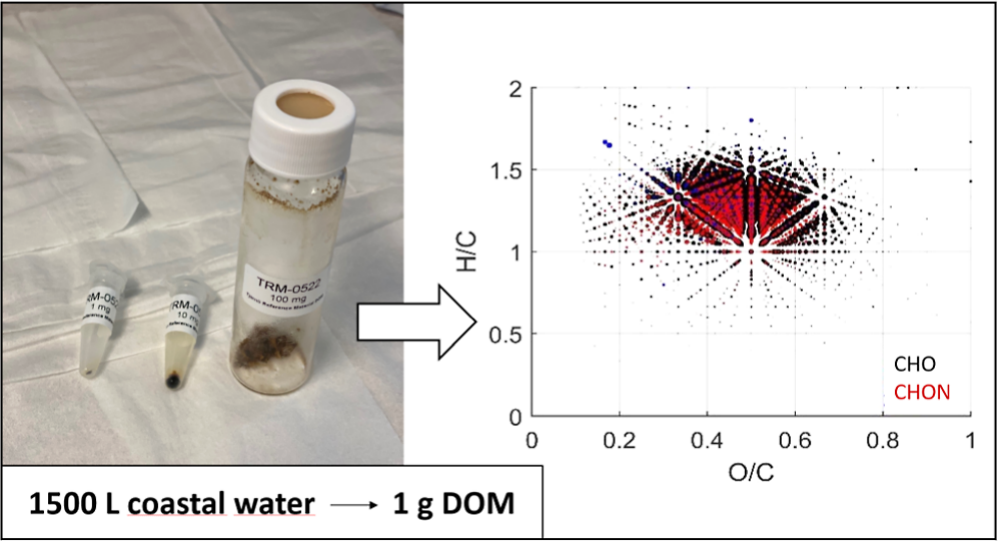

Recent methodological advances have greatly increased our ability to characterize aquatic dissolved organic matter (DOM) using high-resolution instrumentation, including nuclear magnetic resonance (NMR) and mass spectrometry (HRMS). Reliable DOM reference materials are required for further method development and data set alignment but do not currently exist for the marine environment. This presents a major limitation for marine biogeochemistry and related fields, including natural product discovery. To fill this resource gap, we have prepared a coastal marine DOM reference material (TRM-0522) from 45 m deep seawater obtained ∼1 km offshore of Sweden’s west coast. Over 3000 molecular formulas were assigned by direct infusion HRMS, confirming sample diversity, and the distribution of formulas in van Krevelen space was typical for a marine sample, with the majority of formulas in the region H/C 1–1.5 and O/C 0.3–0.7. The extracted DOM pool was more nitrogen (N)- and sulfur (S)-rich than a typical terrestrial reference material (SRFA). MZmine3 processing of ultrahigh-performance liquid chromatography (UPLC)-HRMS/MS data revealed 494 resolvable features (233 in negative mode; 261 in positive mode) over a wide range of retention times and masses. NMR data indicated low contributions from aromatic protons and, generally speaking, low lignin, humic, and fulvic substances associated with terrestrial samples. Instead, carboxylic-rich aliphatic molecules were the most abundant components, followed by carbohydrates and aliphatic functionalities. This is consistent with a very low specific UV absorbance SUVA_254_ value of 1.52 L mg C^–1^ m^–1^. When combined with comparisons with existing terrestrial reference materials (Suwannee River fulvic acid and Pony Lake fulvic acid), these results suggest that TRM-0522 is a useful and otherwise unavailable reference material for use in marine DOM biogeochemistry.

## 1 Introduction

Dissolved organic matter (DOM) is a complex mixture of thousands if not millions of compounds.^1,2^ It is ubiquitous in aquatic systems, where it underpins food webs, plays a critical role in the global carbon cycle,^3^ and may hold a wealth of natural products for use in biotechnology applications.^4^ It is therefore critical that researchers have the means and understanding with which to adequately quantify and characterize DOM, but its highly dilute and variable nature means that it is challenging to study. As a result, DOM chemical composition remains poorly characterized.^5−7^

In recent years, several advanced methods have been developed which have allowed progress to be made, such as those using nuclear magnetic resonance (NMR) and high-resolution mass spectrometry (HRMS), including tandem mass spectrometry (HRMS/MS) and coupling to ultrahigh-performance liquid chromatography (UPLC).^8−12^ Complex but reliable reference materials are necessary in order to compare such methods and, critically, to evaluate the scope and implications of the resulting data sets that are generated. Typically, materials from the International Humic Substances Society (IHSS) are used, which are long-established, internationally known and trusted, and can be purchased on the scale of hundreds of milligrams. The main downside of these materials is that they are all sourced from terrestrial, often very brown water environments, with the exception of Pony Lake fulvic acid (PLFA) which came from an Antarctic lake with no higher-order plants but which has now been depleted with no plans to restock (that we are aware of). The introduction of a marine DOM reference material has been discussed,^13^ and a marine reference for dissolved organic carbon (DOC) concentration analysis has been used successfully for some time^14^ but is not available at sufficient concentrations or volumes as to be useful as a DOM characterization standard. Thus, a suitable reference material for marine DOM studies does not currently exist. The lack of a marine DOM reference material is problematic and hinders method development and data set alignment in marine biogeochemistry within the DOM characterization field and in related subjects such as natural product discovery and trace metal speciation analysis.

In order to fill this resource gap, we have prepared and characterized a marine DOM reference material from 45 m deep seawater obtained from the Tjärnö Marine Laboratory (Gothenburg University) on the west coast of Sweden, which is representative of a coastal marine environment. The material was collected in this first effort in May 2022 from 1500 L of seawater using a commercially available aqueous-compatible C18 column (C18-AQ hereafter), and after sample clean-up, totaled 1.06 g in mass. We have given the sample a unique designation “Tjarno Reference Material—May 2022” (TRM-0522), allowing for future efforts to collect and distribute more material. In this article, we characterize the material using absorbance, fluorescence, HRMS, UPLC-HRMS/MS, and NMR.

## 2 Methods

### 2.1 Reagents

LC–MS-grade methanol (Supelco LiChroSolv hypergrade for LC–MS) and fuming hydrochloric acid (37% HCl) were obtained from Merck (UK). Acetonitrile (ACN) was obtained from Sigma-Aldrich (Supelco LiChroSolv hypergrade for LC–MS), formic acid (FA) was obtained from VWR (AmalaR Normapur, VWR Sweden), and 25% ammonia (NH_3_) was obtained from Sigma-Aldrich (Merck Life Science AB, Sweden, Supelco Suprapur grade). Hippuric acid, fuscidic acid, chloroform (CHCl_3_) containing 100–200 ppm amylenes as the stabilizer, deuterium oxide (D_2_O), and 25% ammonium hydroxide (NH_4_OH) solution were obtained from Sigma-Aldrich (Merck Life Science AB, Sweden). Suwannee River fulvic acid (SRFA; batch number 2S101F) and PLFA (batch number 1R109F) were obtained from the IHSS (Saint-Paul, USA).

### 2.2 DOM Extraction

Seawater was obtained from the Tjärnö Marine Laboratory seawater system (Text S1), which draws water from a 45 m deep intake located ∼1.0 km off-shore of the nearest land mass on the west coast of Sweden at 58°52.843 N 11°06.378 E (Figure S1).

**Figure 1:**
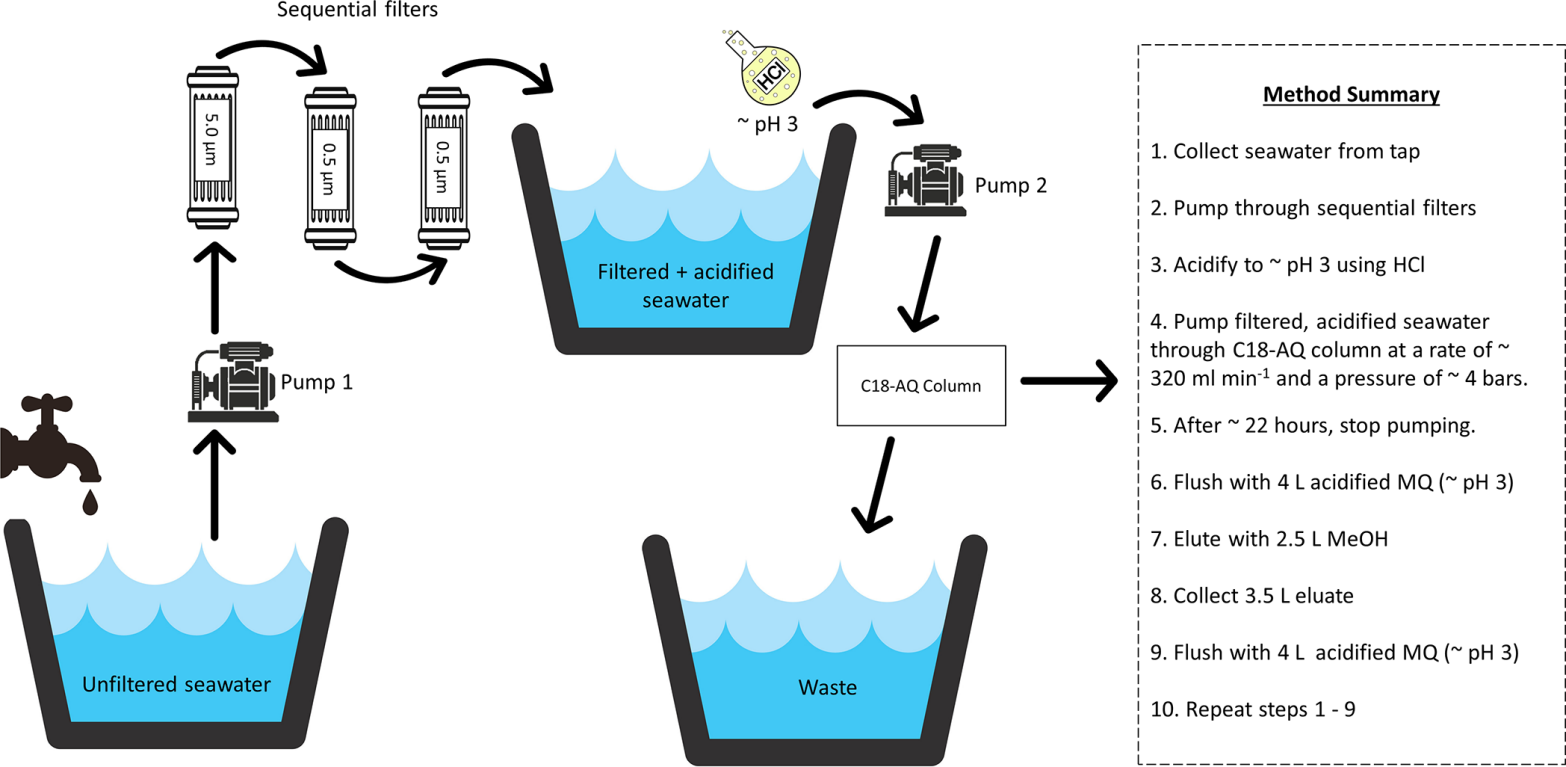
Schematic diagram and method summary describing the DOM extraction method, from collection of seawater from the tap to daily column elution.

The DOM extraction process is summarized in Figure 1. Each day, a fresh batch of seawater was collected from the tap into a clean 1000 L HDPE aquarium tank. From there, it was pumped sequentially through three pool filters (5, 0.5, and 0.5 μm pore size) into a second clean 1000 L HDPE tank, where it was acidified with hydrochloric acid (HCl) to a pH of ∼3. This water was pumped through a preconditioned (Text S2) PuriFlash C18-AQ F1600 flash column (Interchim) for ∼22 h each day. Accumulated salts were then removed by flushing the column with ∼2 column volumes (4 L) acidified MilliQ. The column was then flushed with a further 4 L acidified MilliQ water. During the elution process, a fresh batch of seawater was filtered and acidified, after which the process was repeated.

Eluted MeOH was collected into a series of ashed (4 h at 550 °C) glass Duran bottles and chilled overnight, along with a portion of acidified MilliQ which came through the column on either side of MeOH (∼3.5 L total each day). The reason we collected the MilliQ fraction was to ensure that no MeOH (and by extension, no retained DOM) was missed. A total of 1500 L of coastal seawater was pumped through the column over 5 days.

### 2.3 Sample Processing

Each eluted sample was dried down into a round-bottom flask using a rotary evaporator, and the dried material was transferred into an 8 mL amber glass vial using small volumes of ACN and ultrapure water (MilliQ). A residue which could not be dissolved in ACN or ultrapure water was removed using 50% MeOH, which was then removed using a rotary evaporator. We suspected that this residue was the leached column material and so additional processing steps (mostly liquid–liquid extraction between basic water and chloroform) were added to ensure its complete removal. Details of these steps are given in Text S3 and resulted in a dried DOM mass of 1058 mg. The dried DOM was redissolved in 100 mL of 10% MeOH in MilliQ water with 0.1% NH_3_, aliquoted for distribution, and dried down using a Speedvac (1 and 10 mg of aliquots) or freeze drier (larger aliquots). A 1 mg subsample was rediluted in 41 mL of MilliQ water, giving a DOM concentration of 24.7 mg L^–1^. DOC concentration was 10.1 mg L^–1^, equivalent to 41% DOM. Aliquots were stored in a - 20 °C freezer in 1.5 mL Eppendorf tubes (1 and 10 mg of aliquots), 8 mL amber glass vials (20 mg of aliquots), and 40 mL clear glass vials (50 and 100 mg of aliquots).

### 2.4 Analysis

#### 2.4.1 DOC, Absorbance, and Fluorescence

DOC was measured using a Sievers M9 TOC analyzer and quantified as nonpurgeable organic carbon against MilliQ blanks and a potassium hydrogen phthalate (KPH) standard. Replicate measurements had a coefficient of variation of ≤2%. The instrument was calibrated between 1 and 50 mg L^–1^ using KPH, and subsequent KPH standards were all within 5% of their calibrated value.

Absorbance was measured between 200 and 600 nm at 1 nm intervals and with a 240 nm min^–1^ scan speed and a 2 nm slit width using a Lambda 40 UV–vis spectrophotometer (PerkinElmer, Waltham, MA) and a 1 cm pathlength quartz cuvette. Samples were blank corrected against MilliQ water. DOC specific absorbance at 254 nm (SUVA_254_), a commonly used metric for aromaticity where a higher number is associated with a greater aromatic content, was calculated by dividing absorbance at 254 nm (in cm^–1^) by DOC concentration (in mg L^–1^) and multiplying by 100.^15^ Spectral slopes between 275 and 295 nm (S_275–295_) and 350 and 400 (S_350–400_) and the ratio between the two (SR) were also calculated.^16^ S_275–295_ and SR are both inversely related to average DOM molecular weight.

Fluorescence was measured at excitation (ex) wavelengths between 250 and 445 nm at 5 nm intervals and emission (em) wavelengths between 300 and 600 nm at 4 nm increments using a Horiba Fluoromax-4 spectrophotometer (Horiba, Kyoto, Japan) in ratio mode and a 1 cm pathlength quartz cuvette. Slit widths were set to 5 nm, with a 0.1 s integration time. The resultant excitation-emission matrix (EEM) was blank corrected using the EEM of MilliQ water, corrected for inner-filter effects,^17^ and normalized to Raman units.^18^ Common fluorescence indices [humification (HI),^19^ fluorescence (FI),^20^ and diagenetic freshness (β/α)^21^ were determined alongside Coble peaks,^22^ which give a broad characterization of compound class (e.g., humic-like; protein-like; and marine). All corrections and calculations were performed using the FDOMcorr toolbox^23^ for MATLAB.^24^

#### 2.4.2 High-Resolution Mass Spectrometry

Dried samples were accurately weighed and dissolved/diluted to 50 mg DOM L^–1^ with 50% methanol (LCMS grade, LiChroSolv, VWR) in ultrapure water (Milli-Q, Millipore). These solutions were infused at 10 μL min^–1^ into a high-resolution mass spectrometer (Q-Exactive Orbitrap, Thermo Fisher) equipped with a heated electrospray ionization source operating in negative ion mode at 100 °C with a setting of 25 units of sheath gas and 3 kV. The S-lens was set to 60, and capillary temperature was 250° °C. The MS resolution was set to 140,000 at 200 Da, and transients were measured between *m*/*z* 150 and 1000, with 369.11911 and 525.19775 sets as lock masses. 500 transients were collected and averaged, and a wash solution of 50% methanol: water (LCMS grade) was infused between samples until the blank signal stabilized. Formulas were assigned allowing 4–50 C, 4–100 H, 2–40 O, 0–2 N, 0–1 S, and 0–1 ^13^C. Combinations of N, S, and ^13^C were not allowed nor were double-bond equivalence minus O greater than 10. A 5th order polynomial calibration was performed using a series of expected ions, as given in a previous paper,^25^ and formulas were assigned if the mass offset was less than 1.5 ppm.

#### 2.4.3 UPLC–High-Resolution Tandem Mass Spectrometry

A 1 mg dried aliquot was dissolved in 200 μL of 10% LCMS-grade methanol in LCMS-grade water, sonicated for 5 min, and vortexed to give a concentration of 5000 mg L^–1^. 10 μl of the sample containing 50 μg of DOM was injected onto a UPLC C18 column (2 × 150 mm, 1.7 μm, Phenomenex Kinetix Core Shell) at a flow rate of 0.4 mL min^–1^ using a Vanquish UPLC (Thermo Fisher). Three mobile phases were prepared: LCMS-grade water with 0.1% FA (A), LCMS-grade acetonitrile with 0.1% FA (B), and 90:10 LCMS-grade MeOH in LCMS-grade water with 0.05% FA and 0.25% NH_3_. The gradient was run as follows: 0–1 min = 1% B, 0% C; 1–9 min = 1–99% B, 0% C; 9–10 min = 99% B, 0% C; 10–10.1 min = 99–1% B, 0–99% C; 10.1–11.4 min = 1% B, 99% C; 11.4–11.5 min = 1% B, 99–0% C; and 11.5–15 min = 1% B, 0% C. An Orbitrap Q Exactive mass spectrometer was used, operating in negative mode for one analysis (triplicates plus blank) and positive mode for another (triplicates plus blank). Data were collected in a data-dependent mode, with the top five peaks sent for HCD fragmentation, and are available at the MassIVE data repository (https://massive.ucsd.edu/; MSV000090282). MZmine3 was used to align MS1-level LC–MS features^26^ and find chromatographically resolved peaks^10^ (MZmine3 project available in the Supporting Information), and Global Natural Product Social Molecular Networking (GNPS) jobs were run on positive and negative mode fragmentation data^27^ to cluster and library match fragmentation patterns at the MS2 level.^28^

#### 2.4.4 Nuclear Magnetic Resonance

NMR spectra were acquired at 298 K on a Bruker 500 MHz spectrometer using a TXO CRPHe TR-^13^C/^15^N/^1^H 5 mm-Z CryoProbe. The samples were dissolved in D_2_O and referenced to the residual solvent peak at 4.79 ppm in the ^1^H NMR spectrum or to MeOH at 49.50 ppm which was added as an external reference for the ^13^C NMR spectrum. ^1^H NMR spectra were gathered over 330 scans, with a 3.28 s acquisition time and a 15.3 s relaxation delay. ^13^C NMR spectra were gathered over 9600 scans, with a 0.92 s acquisition time and an 8 s relaxation delay.

## 3 Results and Discussion

### 3.1 [DOM], [DOC], Absorbance, and Fluorescence

The final dry DOM mass was 1.06 g, equivalent to 0.71 g of DOM L^–1^ seawater extracted. DOC accounted for 41% of the extracted DOM, equivalent to 0.29 g of DOC L^–1^ seawater extracted (24.2 μM SPE-DOC). DOC typically comprises ∼50% DOM in humics^29^ which are likely to be highly phenolic and aromatic in character and ∼45% in freshwaters, where humics are mixed with the rest of the aquatic DOM pool.^30^ The aged, recalcitrant DOM typically found in seawater is thought to be dominated by alicyclic and linear compounds which are rich in carboxylic acids,^6,31^ and the increased abundance of oxygen from carboxyl groups likely decreases the percentage of carbon in marine DOM relative to the terrestrial systems.

Absorbance at 254 nm was 0.152 cm^–1^, giving a SUVA_254_ of 1.52 L mg C^–1^ m^–1^. SUVA_254_ typically ranges from 1.0 to 4.0 L mg C^–1^ m^–1^ in surface waters,^32^ although values >6.0 L mg C^–1^ m^–1^ have been reported for samples with a strong terrestrial signal^33^ or with a high iron content,^34^ and values <1 L mg C^–1^ m^–1^ have been reported for samples dominated by fresh DOM production (e.g., algal leachate samples^35^). Coastal ocean samples typically exhibit values of around 1.5–3.5 L mg C^–1^ m^–1^, while open ocean samples tend to be closer to 1.0 L mg C^–1^ m^–1^.^36^ Thus, the SUVA_254_ value of TRM-0522 (1.52 L mg C^–1^ m^–1^) is within the expected range and indicates a considerably lower degree of aromaticity relative to other available reference materials, e.g., Suwannee River natural organic matter (SRNOM; 5.34 L mg C^–1^ m^–1^^37^), SRFA (4.20 mg C^–1^ m^–1^^38^), and PLFA (2.0–3.2 L mg C^–1^ m^–1^^39^). S_275–295_ and S_350–400_ were 0.18 and 0.17, respectively, giving an SR of 1.05, which is lower than SR typically reported for marine samples, for example, Helms et al. (2008) reported SR values of 9.4 for the Sargasso Sea, dropping to 3.9 on the continental slope and 1.5 at the shelf break, relative to SRNOM which has an SR of around 0.70.^16^ The SR of TRM-0522 describes the extracted rather than the bulk sample, and so an SR of 1.05 suggests that the extracted DOM fraction is biased toward higher-molecular-weight compounds. An absorbance spectrum and EEM for TRM-0522 diluted to a concentration of 10.1 mg L^–1^ are shown in Figure S3, with the associated Coble peaks listed in Table S1. TRM-0522 fluoresces highly in the peak T and M regions, indicative of protein-like and marine humic-like DOM, with a smaller but notable contribution of peak A, terrestrial humic-like fluorescence. FI, HIX, and β/α were 1.49, 1.59, and 1.67, respectively. FI and HIX are indicative of the degree of autochthonous and humified material in a sample and together indicate that TRM-022 contains DOM with a low degree of humification which is typical of marine samples.^40^ β/α provides a measure of diagenetic status, with higher values indicating less diagenetically altered (i.e. “fresher”) DOM, and has previously been reported as < 0.7 in freshwaters^41^ and between 0.65 and 1.3 in coastal waters,^42^ which indicates that TRM-022 contains DOM which is less diagenetically altered than is typical.

### 3.2 High-Resolution Mass Spectrometry

**Figure 2:**
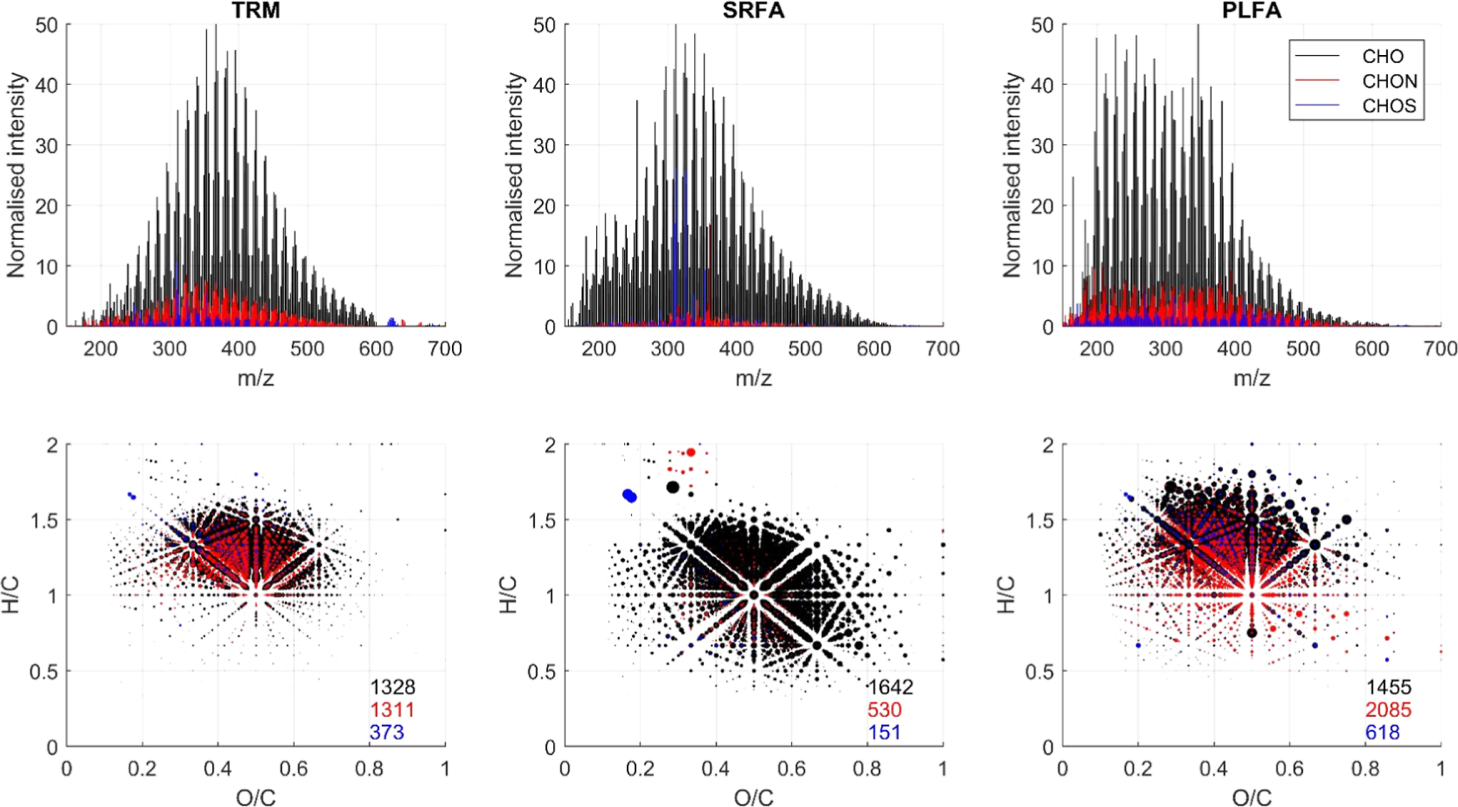
Reconstructed mass spectra (top) and van Krevelen diagrams (bottom) of formulas assigned in the TRM-0522 sample, along with two other reference mixtures from the IHSS—SRFA and PLFA. CHO-containing formulas without other heteroatoms are shown in black, those containing nitrogen (CHON) are shown in red, and those containing sulfur (CHOS) are shown in blue. The total number of each is indicated in the associated van Krevelen diagram.

HRMS analysis yielded >3000 peaks (Table 1). In terms of distribution between CHO-, CHON-, and CHOS-containing formulas, the sample was somewhere between the IHSS reference materials SRFA and PLFA. TRM-0522 contained fewer N- and S-containing peaks than PLFA (which are actually the most numerous in that sample) but was much more N- and S-rich than SRFA (Figure 2). However, it should be noted that multiple N- and S-containing peaks, as well as CHONS peaks, could not be confidently assigned following Orbitrap analysis,^25^ and so the true diversity of the TRM-0522 sample could not be completely assessed.^43^

**Table 1:** HRMS Peak Metrics for TRM-0522, SRFA, and PLFA Showing the Number of Peaks (Peaks) and the Intensity-Weighted Average of Oxygen to Carbon (O/C_wa_), Hydrogen to Carbon (H/C_wa_), and Mass to Charge (*m*/*z*_wa_) Ratios and the Number of Common Peaks Detected Relative to a Recent Interlaboratory Study^25^

	peaks	O/C_wa_	H/C_wa_	m/z_wa_	Interlab common peaks
TRM-0522	3012	0.46	1.28	385.8	N/A
SRFA (2S101F)	2323	0.50	1.10	359.3	92%
PLFA (1R109F)	4158	0.43	1.33	322.7	95%

The distribution of formulas in van Krevelen space was typical for a coastal marine sample,^7,44^ with the majority of formulas in the region H/C 1–1.5 and O/C 0.3–0.7 (Figure 2). This region is where the most recalcitrant DOM is typically found, namely, “Island of Stability” molecules^45^ and CRAM formulas.^46^ These data are consistent with the NMR findings (Section 3.4) and suggest that the TRM sample is a useful and otherwise unavailable reference mixture for relatively aged, recalcitrant marine DOM.

### 3.3 UPLC–High-Resolution Tandem Mass Spectrometry

**Figure 3:**
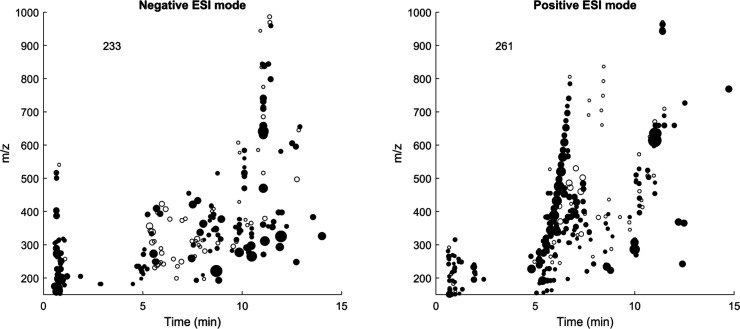
UPLC-HRMS features determined by MZmine3, shown as *m*/*z* vs retention time, and with relative intensity in triplicate analysis shown as point size. Only points with an average more than 10× blank are shown, and their number is indicated in text at the top left. Filled-in features are those which triggered a data-dependent analysis and for which fragmentation mass spectra are available.

UPLC–HRMS/MS analysis allows a greater level of molecular detail to be inspected by separating the geochemical DOM background across a wide elution period, albeit without finely detailed chromatographic resolution,^8,10^ and enabling determination and annotation of truly resolvable metabolite features.^11,28^ A variety of resolvable features were found over a wide range of retention times and masses (Table S2), numbering 233 (negative) and 261 (positive). Unlike the typical broadly eluting features found in the geochemically degraded DOM background, these features are well resolved and can be used as reference peaks for method development, validation, and drift correction if TRM-0522 is included in metabolomic data sets. The number of library hits found using GNPS molecular networking was surprisingly low (only five per ionization mode and most of these known laboratory contaminants). This low number of hits may be due to the fact that so much of DOM is not characterized and annotated^47^ and may also be due to the extraction method used here being poor at retaining particularly hydrophilic compounds. Possibly, the pipeline and gravel filtration method used to collect coastal water for the aquarium activities at the research site also filters out many known metabolites. However, hundreds of resolvable features were found at the MS1 and MS2 level (Figure 3); they simply were not matched to library compounds.

### 3.4 Nuclear Magnetic Resonance

**Figure 4:**
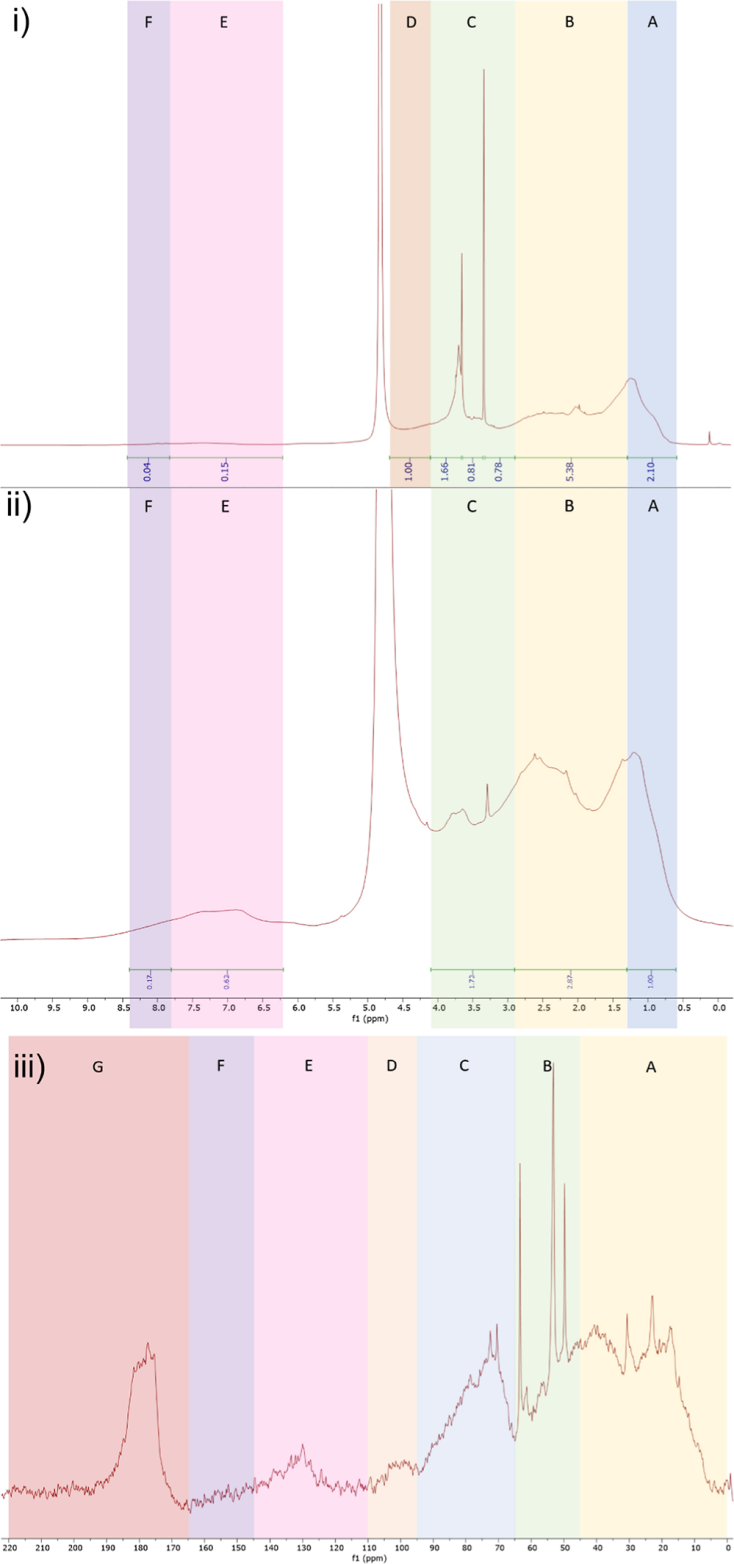
(i) ^1^H NMR spectrum of TRM-0522 at 500 MHz in D_2_O and (ii) ^1^H NMR spectrum of SRFA at 500 MHz in D_2_O. Coloured boxes A–F indicate defined regions corresponding to different groups. (iii) ^13^C NMR spectrum of TRM-0522 at 125 MHz in D_2_O. Colored boxes A–G indicate defined regions corresponding to different groups.

By examining the ^1^H NMR spectrum, six distinct regions can be identified (Figure 4), with ranges for these regions defined by previous work.^48^ Region A spans from 0.6 to 1.3 ppm and corresponds to aliphatic polymethylene and methyl functionalities, while region B, spanning from 1.3 to 2.9 ppm, includes *N*- and *O*-substituted aliphatic signals. Further downfield, region C includes predominantly *O*-alkyl signals and covers 2.9–4.1 ppm. Region D is composed predominantly of the α-proton of peptides, comprising signals from 4.1 to 4.8 ppm. The presence of the residual water peak at 4.79 ppm obscures any accurate integration of the anomeric protons of carbohydrates. Aromatic and phenolic hydrogen atoms can be observed between 6.2 and 7.8 ppm in region E. Finally, amidic protons can typically be observed in region F from 7.8 to 8.4 ppm. However, exchange with deuterium from the solvent means this signal is nonquantitative. The peak observed at 3.36 ppm is attributable to residual methanol. While a strong resonance indicative of methoxy ethers is seen from 3.6 to 3.8 ppm, no peak at 6.57 ppm, typically attributable to the aromatic protons of lignin, is observed.^12^ Similar methoxy ether peaks have been reported in Atlantic surface water^6^ at all sampled depths (5, 48, 200, and 5446 m), with relatively minor variations in intensity. We cannot rule out that this peak is derived from lignin in TRM-0522, but the lack of aromatic C–H protons attributable to lignin makes it difficult to conclusively state that this methoxy ether peak is lignin derived. The relative amounts of each accurately definable area (regions A–E) are presented in Table 2.

**Table 2:** Functionalities Related to and Percentage Contribution of Integrals for Each Region in the ^1^H NMR Spectrum of TRM-0522 and SRFA (with Adjusted TRM-0522 Contribution in Brackets)

region	ppm range	functionalities	% contribution
TRM-0522			
A	0.6–1.3	polymethylene, methyl	18
B	1.3–2.9	N- and O-substituted aliphatics	45
C	2.9–4.1	O-alkyl	27
D	4.1–4.8	peptide α-proton	9
E	6.2–7.8	aromatic and phenolic	1
SRFA			
A	0.6–1.3	polymethylene, methyl	16 (19)
B	1.3–2.9	N- and O-substituted aliphatics	46 (49)
C	2.9–4.1	O-alkyl	28 (31)
E	6.2–7.8	aromatic and phenolic	10 (1)

The ^1^H NMR spectra of TRM-0522 retain the key features that have previously been described for marine and freshwater DOM.^31,49,50^ Specifically, regions have been attributed to carbohydrates and aliphatic compounds.^31^ The aliphatic region has been described as including both peptide-derived aliphatics but also material derived from linear terpenoids (MDLT).^51^ Roughly, the ranges displayed on Figure 4 and Table 2 correlate with these constituent molecules, with range A including aliphatics, range B including CRAM, and range C including carbohydrates. While the percentage contributions obtained by region integration can only approximately estimate the actual compound composition, the integrated ranges are in alignment with previously reported data that show CRAM as the most abundant component, followed by carbohydrates and finally aliphatics.^49^ Of note is a pronounced feature at 3.52–3.76 ppm, which in the majority of previously reported samples is not observed as a distinct peak.^31,49,50^ However, this peak is seen in spectra of samples isolated at several depths in the Atlantic ocean^31^ and is possibly derived from methoxy-ether functionalities.^6^ Consistent with other marine water samples, TRM-0522 lacks strong signals attributable to alkenes or aromatics, indicating a low contribution from lignin, humic, and fulvic substances. This also corresponds well to our finding of very low SUVA_254_ (1.52 L^–1^ g cm^–1^).

In the ^13^C NMR spectrum (Figure 4), seven separate regions are presented, according to definitions used in previous reports.^52−56^ Region A covers 0–45 ppm and contains signals corresponding to alkyl chains that are not functionalized with heteroatoms. From 45 to 65 ppm, region B presents substituted alkyl carbons attached predominantly to nitrogen atoms but also some oxygen atoms (e.g. methoxy carbons), as well as some highly branched aliphatic signals that neighbor, but are not directly attached to, heteroatoms. Region C, from 65 to 95 ppm, contains predominantly signals attributable to oxygen-substituted carbon atoms, such as ethers and ring carbons in carbohydrate molecules. Region D, from 95 to 110 ppm, contains dioxygenated carbons, such as those from the anomeric position of carbohydrates. Next, region E consists of aromatic carbon atom signals and covers the range of 110–145 ppm, while region F consists of phenolic carbon atom signals and spans 145–165 ppm. Finally, signals for carboxylic functionalities, including acids, amides, esters, and ketones, are found in region G, covering 165–220 ppm. A peak corresponding to residual methanol can be observed at 49.00 ppm.

Generally, information extrapolated from the ^13^C NMR spectrum of TRM-0522 agrees with the analogous ^1^H NMR spectrum. Furthermore, the general regions and rough peak shapes correspond to previously reported spectra.^6,31,51,57^ While there are observable peaks in the aromatic/olefinic region, they are relatively minor when compared to those derived from saturated carbon functionalities. This is consistent with the relatively low content of lignin, humic, and fulvic substances in oceanic samples.^6,31,51,57^ A carbonyl signal is observed in the region typically associated with carboxylic acid and amide functionalities (∼190–170 ppm), while no detectable signal is associated with other carbonyl functionalities (ketones, aldehydes, carbamates, and carbonates). In agreement with the ^1^H NMR spectrum analysis, a strong band corresponding to the alcoholic carbons in sugar compounds (95–75 ppm) is observed. While the anomeric signals are obscured by the residual water peak in the ^1^H NMR spectrum, a peak attributable to the anomeric carbons of carbohydrates is observed in the ^13^C NMR spectrum (∼105–95 ppm). The remaining two regions contain strong aliphatic signals, with the signal reducing in intensity outside the 55–15 ppm range, consistent with the presence of cyclic and linear terpenoid-derived material, with cyclic material tending toward the higher chemical shift range, and linear material tending toward the lower chemical shift range.^31,51^

For ^13^C NMR comparison, data was used from the U.S. geological survey.^58^ The most obvious difference between the ^13^C NMR spectra of the materials is a significant aromatic peak spanning 110–145 ppm in the SRFA spectra, a region in which only relatively minor resonances can be observed in the TRM-0522 spectra. This finding reinforces the lack of the relatively photolabile humic, fulvic, and lignin substances in TRM-0522 and suggests that the material is unlikely to be photoreactive. While other regions in the ^13^C NMR spectra of the two samples are relatively similar, differences are noted with the increased peak height in regions attributable to sugars in TRM-0522 (when compared to aliphatic signals) and an increased peak height indicative of substances attributable to carbonyl functionalities in SRFA (when compared to aliphatic signals).

A useful point of comparison for TRM-0522 is the common reputable reference material SRFA. An integrated ^1^H NMR spectrum of SRFA was obtained (Figure 4) using the same parameters and instrument as were used for TRM-0522. Regions were defined in the same way as that for TRM-0522 (see earlier), but a broadening of the residual water peak (4.79 ppm) led to overlap with region D (typically containing information regarding the α-proton of peptides), thus preventing accurate integration of region D. To account for this in comparison to TRM-0522, adjusted contributions of TRM-0522 are shown that exclude region D, alongside the relative contributions of SRFA (Table 2).

Two key pieces of information can be observed by comparing TRM-0522 with SRFA. First, SRFA contains significantly more material with aromatic hydrogens than TRM-0522 does (10 vs 1%, respectively). Aromatic hydrogens are typically associated with humic and fulvic substances, as well as lignin. In SRFA, these hydrogens should predominantly come from fulvic acid, but the presence of lignin cannot be ruled out as the aromatic window is broad and covers the signature peak for lignin at 6.57 ppm. Second, the ratios of the A, B, and C regions remain almost identical between spectra of SRFA and TRM-0522 samples, suggesting that the relative amounts of MDLT, CRAM, and complex polysaccharides remain consistent between the two samples.

### 3.5 Broader Context

We note ongoing discussion within the IHSS on the need for more diverse DOM standards, including for open ocean marine DOM.^13^ The small cartridge size typically used has thus far presented a barrier to extraction of marine DOM on the scales required to produce meaningful quantities of reference material.^13^ The large-scale extraction method detailed in this article and applied in a coastal setting could be applied to open waters with little need for further development.

Use of the existing marine DOC reference material^14^ as a DOM standard is technically feasible, but we consider this to be an inefficient option: assuming a typical DOC concentration of 0.5 mg L^–1^, a C content of 50%, and an extraction efficiency of 50%; a 1 mg of DOM extraction would require 100 × 20 mL vials at a cost of $140 USD. TRM-022 is currently available at a cost of approximately $25 USD per 1 mg and has been well characterized using several common high-resolution methods (NMR; HRMS: UPLC-HRMS/MS). Thus, TRM-022 fills a resource gap for a marine DOM reference material.

TRM-0522 is not an open ocean marine standard but rather a DOM standard obtained from a coastal marine setting. While it is compositionally very different from available terrigenous standards (i.e., SRFA and PLFA), we do acknowledge some terrigenous input. However, until such time as a fully marine, open water DOM standard can be made available, TRM provides the research community with a reference material which is considerably more similar to oceanic waters than the existing terrigenous standards are. Once a fully marine DOM standard exists, we believe that a coastal marine sample will remain of great value, particularly with regards the growing number of land–ocean studies which must operate across fresh and marine waters. In this context, TRM fills an important niche between a fully terrigenous and a fully marine sample.

## 4 Conclusions

The results presented in this paper demonstrate that TRM-0522 is a useful and reasonably representative reference material for use in marine DOM biogeochemistry, particularly in a coastal setting. TRM-0522 is now available to the research community, and we encourage its use in marine biogeochemical studies including DOM characterization, natural product research, and metabolomics. During our first effort, we have made 700 mg of marine DOM reference material available, with future, larger-scale extractions planned. 50 mg of the current batch is available free of charge for researchers with financial barriers to purchase. A fixed extraction point associated with an established university research laboratory (Tjärnö Marine Laboratory, University of Gothenburg) was selected to ensure ongoing access, consistency of source, and a long-term supply. Thus, we intend to make TRM widely available and encourage its day-to-day analytical use. Enquiries can be sent to the corresponding author (JAH).

## Supporting Information Available

Tjärnö seawater system; column preconditioning; extract purification; map of extraction point; salinity and temperature data from extraction point; absorbance scan and EEM of extract; Coble peaks; and resolvable peak metrics (PDF)
Direct infusion analysis (ZIP)

## Notes

MZmine projects and direct infusion mass spectrometry data sets and data processing code are also available as supplementary files. GNPS MS2-level clustering and library matching are available at the following links: https://gnps.ucsd.edu/ProteoSAFe/result.jsp?task=84f7a8209cd6444f85027aabf8357ad4&view=view_all_clusters_withID_beta#%7B%22table_sort_history%22%3A%22main.LibraryID_asc%22%7D, https://gnps.ucsd.edu/ProteoSAFe/result.jsp?task=e2f9d825b55e49cf91c3f99a2c3ed5a8&view=view_all_clusters_withID_beta#%7B%22main.DefaultGroups_input%22%3A%22G1%22%2C%22table_sort_history%22%3A%22main.DefaultGroups_dsc%22%7D.

## References

[ref1] Zark M.; Christoffers J.; Dittmar T. Molecular Properties of Deep-Sea Dissolved Organic Matter Are Predictable by the Central Limit Theorem: Evidence from Tandem FT-ICR-MS. Mar. Chem. 2017, 191, 9–15. 10.1016/j.marchem.2017.02.005.

[ref2] Hawkes J. A.; Patriarca C.; Sjöberg P. J. R.; Tranvik L. J.; Bergquist J. Extreme Isomeric Complexity of Dissolved Organic Matter Found across Aquatic Environments. Limnol. Oceanogr. Lett. 2018, 3, 21–30. 10.1002/lol2.10064.

[ref3] Nebbioso A.; Piccolo A. Molecular Characterization of Dissolved Organic Matter (DOM): A Critical Review. Anal. Bioanal. Chem. 2013, 405, 109–124. 10.1007/s00216-012-6363-2. 22965531

[ref4] Catalá T. S.; Shorte S.; Dittmar T. Marine Dissolved Organic Matter: A Vast and Unexplored Molecular Space. Appl. Microbiol. Biotechnol. 2021, 105, 7225–7239. 10.1007/s00253-021-11489-3. 34536106 PMC8494709

[ref5] Baltar F.; Alvarez-Salgado X. A.; Arístegui J.; Benner R.; Hansell D. A.; Herndl G. J.; Lønborg C. What Is Refractory Organic Matter in the Ocean?. Front. Mar. Sci. 2021, 8, 1–7. 10.3389/fmars.2021.642637.35685121

[ref6] Hertkorn N.; Harir M.; Koch B. P.; Michalke B.; Schmitt-Kopplin P. High-Field NMR Spectroscopy and FTICR Mass Spectrometry: Powerful Discovery Tools for the Molecular Level Characterization of Marine Dissolved Organic Matter. Biogeosciences 2013, 10, 1583–1624. 10.5194/bg-10-1583-2013.

[ref7] Riedel T.; Dittmar T. A Method Detection Limit for the Analysis of Natural Organic Matter via Fourier Transform Ion Cyclotron Resonance Mass Spectrometry. Anal. Chem. 2014, 86, 8376–8382. 10.1021/ac501946m. 25068187

[ref8] Han L.; Kaesler J.; Peng C.; Reemtsma T.; Lechtenfeld O. J. Online Counter Gradient LC-FT-ICR-MS Enables Detection of Highly Polar Natural Organic Matter Fractions. Anal. Chem. 2020, 93, 1740–1748. 10.1021/acs.analchem.0c04426. 33370097

[ref9] Hertkorn N.; Ruecker C.; Meringer M.; Gugisch R.; Frommberger M.; Perdue E. M.; Witt M.; Schmitt-Kopplin P. High-Precision Frequency Measurements : Indispensable Tools at the Core of the Molecular-Level Analysis of Complex Systems. Anal. Bioanal. Chem. 2007, 389, 1311–1327. 10.1007/s00216-007-1577-4. 17924102 PMC2259236

[ref10] Patriarca C.; Bergquist J.; Sjöberg P. J. R.; Tranvik L.; Hawkes J. A. Online HPLC-ESI-HRMS Method for the Analysis and Comparison of Different Dissolved Organic Matter Samples. Environ. Sci. Technol. 2018, 52, 2091–2099. 10.1021/acs.est.7b04508. 29241333

[ref11] Petras D.; Minich J. J.; Cancelada L. B.; Torres R. R.; Kunselman E.; Wang M.; White M. E.; Allen E. E.; Prather K. A.; Aluwihare L. I.; Dorrestein P. C. Non-Targeted Tandem Mass Spectrometry Enables the Visualization of Organic Matter Chemotype Shifts in Coastal Seawater. Chemosphere 2021, 271, 129450 10.1016/j.chemosphere.2020.129450. 33460888 PMC7969459

[ref12] Woods G. C.; Simpson M. J.; Koerner P. J.; Napoli A.; Simpson A. J. HILIC-NMR: Toward the Identification of Individual Molecular Components in Dissolved Organic Matter. Environ. Sci. Technol. 2011, 45, 3880–3886. 10.1021/es103425s. 21469703

[ref13] Chin Y.-P.; McKnight D. M.; D’Andrilli J.; Brooks N.; Cawley K.; Guerard J.; Perdue E. M.; Stedmon C. A.; Tratnyek P. G.; Westerhoff P.; Wozniak A. S.; Bloom P. R.; Foreman C.; Gabor R.; Hamdi J.; Hanson B.; Hozalski R. M.; Kellerman A.; McKay G.; Silverman V.; Spencer R. G. M.; Ward C.; Xin D.; Rosario-Ortiz F.; Remucal C. K.; Reckhow D. Identification of Next-Generation International Humic Substances Society Reference Materials for Advancing the Understanding of the Role of Natural Organic Matter in the Anthropocene. Aquat. Sci. 2023, 85, 32 10.1007/s00027-022-00923-x.

[ref14] Hansell D. A. Dissolved Organic Carbon Reference Material Program. Eos, Transactions American Geophysical Union 2005, 86, 318 10.1029/2005EO350003.

[ref15] Weishaar J. L.; Aiken G. R.; Bergamaschi B. A.; Fram M. S.; Fujii R.; Mopper K. Evaluation of Specific Ultraviolet Absorbance as an Indicator of the Chemical Composition and Reactivity of Dissolved Organic Carbon. Environ. Sci. Technol. 2003, 37, 4702–4708. 10.1021/es030360x. 14594381

[ref16] Helms J. R.; Stubbins A.; Ritchie J. D.; Minor E. C.; Kieber D. J.; Mopper K. Absorption Spectral Slopes and Slope Ratios as Indicators of Molecular Weight, Source, and Photobleaching of Chromophoric Dissolved Organic Matter. Limnol. Oceanogr. 2008, 53, 955–969. 10.4319/lo.2008.53.3.0955.

[ref17] Kothawala D. N.; Murphy K. R.; Stedmon C. A.; Weyhenmeyer G. A.; Tranvik L. J. Inner Filter Correction of Dissolved Organic Matter Fluorescence. Limnol. Oceanogr.: Methods 2013, 11, 616–630. 10.4319/lom.2013.11.616.

[ref18] Lawaetz A. J.; Stedmon C. A. Fluorescence Intensity Calibration Using the Raman Scatter Peak of Water. Appl. Spectrosc. 2009, 63, 936–940. 10.1366/000370209788964548. 19678992

[ref19] Ohno T. Fluorescence Inner-Filtering Correction for Determining the Humification Index of Dissolved Organic Matter. Environ. Sci. Technol. 2002, 36, 742–746. 10.1021/es0155276. 11878392

[ref20] Cory R. M.; McKnight D. M. Fluorescence Spectroscopy Reveals Ubiquitous Presence of Oxidized and Reduced Quinones in Dissolved Organic Matter. Environ. Sci. Technol. 2005, 39, 8142–8149. 10.1021/es0506962. 16294847

[ref21] Parlanti E.; Wörz K.; Geoffroy L.; Lamotte M. Dissolved Organic Matter Fluorescence Spectroscopy as a Tool to Estimate Biological Activity in a Coastal Zone Submitted to Anthropogenic Inputs. Org. Geochem. 2000, 31, 1765–1781. 10.1016/S0146-6380(00)00124-8.

[ref22] Coble P. G. Characterization of Marine and Terrestrial DOM in Seawater Using Excitation-Emission Matrix Spectroscopy. Mar. Chem. 1996, 51, 325–346. 10.1016/0304-4203(95)00062-3.

[ref23] Murphy K. R.; Butler K. D.; Spencer R. G. M.; Stedmon C. A.; Boehme J. R.; Aiken G. R. Measurement of Dissolved Organic Matter Fluorescence in Aquatic Environments: An Interlaboratory Comparison. Environ. Sci. Technol. 2010, 44, 9405–9412. 10.1021/es102362t. 21069954

[ref24] MATLAB. Version R2017a, 2017.

[ref25] Hawkes J. A.; D’Andrilli J.; Agar J. N.; Barrow M. P.; Berg S. M.; Catalán N.; Chen H.; Chu R. K.; Cole R. B.; Dittmar T.; Gavard R.; Gleixner G.; Hatcher P. G.; He C.; Hess N. J.; Hutchins R. H. S.; Ijaz A.; Jones H. E.; Kew W.; Khaksari M.; Palacio Lozano D. C.; Lv J.; Mazzoleni L. R.; Noriega-Ortega B. E.; Osterholz H.; Radoman N.; Remucal C. K.; Schmitt N. D.; Schum S. K.; Shi Q.; Simon C.; Singer G.; Sleighter R. L.; Stubbins A.; Thomas M. J.; Tolic N.; Zhang S.; Zito P.; Podgorski D. C. An International Laboratory Comparison of Dissolved Organic Matter Composition by High Resolution Mass Spectrometry: Are We Getting the Same Answer?. Limnol. Oceanogr.: Methods 2020, 18, 235–258. 10.1002/lom3.10364.

[ref26] Pluskal T.; Castillo S.; Villar-Briones A.; Orešič M. MZmine 2: Modular Framework for Processing, Visualizing, and Analyzing Mass Spectrometry-Based Molecular Profile Data. BMC Bioinf. 2010, 11, 395 10.1186/1471-2105-11-395. PMC2918584 20650010

[ref27] Wang M.; Carver J. J.; Phelan V. V.; Sanchez L. M.; Garg N.; Peng Y.; Nguyen D. D.; Watrous J.; Kapono C. A.; Luzzatto-Knaan T.; Porto C.; Bouslimani A.; Melnik A. V.; Meehan M. J.; Liu W.-T.; Crüsemann M.; Boudreau P. D.; Esquenazi E.; Sandoval-Calderón M.; Kersten R. D.; Pace L. A.; Quinn R. A.; Duncan K. R.; Hsu C.-C.; Floros D. J.; Gavilan R. G.; Kleigrewe K.; Northen T.; Dutton R. J.; Parrot D.; Carlson E. E.; Aigle B.; Michelsen C. F.; Jelsbak L.; Sohlenkamp C.; Pevzner P.; Edlund A.; McLean J.; Piel J.; Murphy B. T.; Gerwick L.; Liaw C.-C.; Yang Y.-L.; Humpf H.-U.; Maansson M.; Keyzers R. A.; Sims A. C.; Johnson A. R.; Sidebottom A. M.; Sedio B. E.; Klitgaard A.; Larson C. B.; Boya P C. A.; Torres-Mendoza D.; Gonzalez D. J.; Silva D. B.; Marques L. M.; Demarque D. P.; Pociute E.; O’Neill E. C.; Briand E.; Helfrich E. J. N.; Granatosky E. A.; Glukhov E.; Ryffel F.; Houson H.; Mohimani H.; Kharbush J. J.; Zeng Y.; Vorholt J. A.; Kurita K. L.; Charusanti P.; McPhail K. L.; Nielsen K. F.; Vuong L.; Elfeki M.; Traxler M. F.; Engene N.; Koyama N.; Vining O. B.; Baric R.; Silva R. R.; Mascuch S. J.; Tomasi S.; Jenkins S.; Macherla V.; Hoffman T.; Agarwal V.; Williams P. G.; Dai J.; Neupane R.; Gurr J.; Rodríguez A. M. C.; Lamsa A.; Zhang C.; Dorrestein K.; Duggan B. M.; Almaliti J.; Allard P.-M.; Phapale P.; Nothias L.-F.; Alexandrov T.; Litaudon M.; Wolfender J.-L.; Kyle J. E.; Metz T. O.; Peryea T.; Nguyen D.-T.; VanLeer D.; Shinn P.; Jadhav A.; Müller R.; Waters K. M.; Shi W.; Liu X.; Zhang L.; Knight R.; Jensen P. R.; Palsson B. Ø.; Pogliano K.; Linington R. G.; Gutiérrez M.; Lopes N. P.; Gerwick W. H.; Moore B. S.; Dorrestein P. C.; Bandeira N. Sharing and Community Curation of Mass Spectrometry Data with Global Natural Products Social Molecular Networking. Nat. Biotechnol. 2016, 34, 828–837. 10.1038/nbt.3597. 27504778 PMC5321674

[ref28] Petras D.; Phelan V. V.; Acharya D.; Allen A. E.; Aron A. T.; Bandeira N.; Bowen B. P.; Belle-Oudry D.; Boecker S.; Cummings D. A.; Deutsch J. M.; Fahy E.; Garg N.; Gregor R.; Handelsman J.; Navarro-Hoyos M.; Jarmusch A. K.; Jarmusch S. A.; Louie K.; Maloney K. N.; Marty M. T.; Meijler M. M.; Mizrahi I.; Neve R. L.; Northen T. R.; Molina-Santiago C.; Panitchpakdi M.; Pullman B.; Puri A. W.; Schmid R.; Subramaniam S.; Thukral M.; Vasquez-Castro F.; Dorrestein P. C.; Wang M. GNPS Dashboard: Collaborative Exploration of Mass Spectrometry Data in the Web Browser. Nat. Methods 2022, 19, 134–136. 10.1038/s41592-021-01339-5. 34862502 PMC8831450

[ref29] Nissenbaum A. Phosphorus in Marine and Non-Marine Humic Substances. Geochim. Cosmochim. Acta 1979, 43, 1973–1978. 10.1016/0016-7037(79)90009-7.

[ref30] Moody C. S.; Worrall F. Modeling Rates of DOC Degradation Using DOM Composition and Hydroclimatic Variables. J. Geophys. Res.: Biogeosci. 2017, 122, 1175–1191. 10.1002/2016JG003493.

[ref31] Hertkorn N.; Benner R.; Frommberger M.; Schmitt-Kopplin P.; Witt M.; Kaiser K.; Kettrup A.; Hedges J. I. Characterization of a Major Refractory Component of Marine Dissolved Organic Matter. Geochim. Cosmochim. Acta 2006, 70, 2990–3010. 10.1016/j.gca.2006.03.021.

[ref32] Hansen A. M.; Kraus T. E. C.; Pellerin B. A.; Fleck J. A.; Downing B. D.; Bergamaschi B. A. Optical Properties of Dissolved Organic Matter (DOM): Effects of Biological and Photolytic Degradation. Limnol. Oceanogr. 2016, 61, 1015–1032. 10.1002/lno.10270.

[ref33] Jaffé R.; McKnight D.; Maie N.; Cory R.; McDowell W. H.; Campbell J. L. Spatial and Temporal Variations in DOM Composition in Ecosystems: The Importance of Long-Term Monitoring of Optical Properties. J. Geophys. Res.: Biogeosci. 2008, 113, G04032 10.1029/2008JG000683.

[ref34] Poulin B. A.; Ryan J. N.; Aiken G. R. Effects of Iron on Optical Properties of Dissolved Organic Matter. Environ. Sci. Technol. 2014, 48, 10098–10106. 10.1021/es502670r. 25084347

[ref35] Pellerin B. A.; Hernes P. J.; Saraceno J.; Spencer R. G. M.; Bergamaschi B. A. Microbial Degradation of Plant Leachate Alters Lignin Phenols and Trihalomethane Precursors. J. Environ. Qual. 2010, 39, 946–954. 10.2134/jeq2009.0487. 20400590

[ref36] D’Andrilli J.; Chanton J. P.; Glaser P. H.; Cooper W. T. Characterization of Dissolved Organic Matter in Northern Peatland Soil Porewaters by Ultra High Resolution Mass Spectrometry. Org. Geochem. 2010, 41, 791–799. 10.1016/j.orggeochem.2010.05.009.

[ref37] Palma D.; Khaled A.; Sleiman M.; Voyard G.; Richard C. Effect of UVC Pre-Irradiation on the Suwannee River Natural Organic Matter (SRNOM) Photooxidant Properties. Water Res. 2021, 202, 117395 10.1016/j.watres.2021.117395. 34273776

[ref38] Leresche F.; Torres-Ruiz J. A.; Kurtz T.; von Gunten U.; Rosario-Ortiz F. L. Optical Properties and Photochemical Production of Hydroxyl Radical and Singlet Oxygen after Ozonation of Dissolved Organic Matter. Environ. Sci.: Water Res. Technol. 2021, 7, 346–356. 10.1039/D0EW00878H.

[ref39] Cawley K. M.; McKnight D. M.; Miller P.; Cory R.; Fimmen R. L.; Guerard J.; Dieser M.; Jaros C.; Chin Y.-P.; Foreman C. Characterization of Fulvic Acid Fractions of Dissolved Organic Matter during Ice-out in a Hyper-Eutrophic, Coastal Pond in Antarctica. Environ. Res. Lett. 2013, 8, 045015 10.1088/1748-9326/8/4/045015.

[ref40] D’Andrilli J.; Silverman V.; Buckley S.; Rosario-Ortiz F. L. Inferring Ecosystem Function from Dissolved Organic Matter Optical Properties: A Critical Review. Environ. Sci. Technol. 2022, 56, 11146–11161. 10.1021/acs.est.2c04240. 35917372 PMC9387109

[ref41] Wilson H. F.; Xenopoulos M. A. Effects of Agricultural Land Use on the Composition of Fluvial Dissolved Organic Matter. Nat. Geosci. 2009, 2, 37–41. 10.1038/ngeo391.

[ref42] Lu Y.; Edmonds J. W.; Yamashita Y.; Zhou B.; Jaegge A.; Baxley M. Spatial Variation in the Origin and Reactivity of Dissolved Organic Matter in Oregon-Washington Coastal Waters. Ocean Dynam. 2015, 65, 17–32. 10.1007/s10236-014-0793-7.

[ref43] Smith D. F.; Podgorski D. C.; Rodgers R. P.; Blakney G. T.; Hendrickson C. L. 21 Tesla FT-ICR Mass Spectrometer for Ultrahigh-Resolution Analysis of Complex Organic Mixtures. Anal. Chem. 2018, 90, 2041–2047. 10.1021/acs.analchem.7b04159. 29303558

[ref44] Seidel M.; Vemulapalli S. P. B.; Mathieu D.; Dittmar T. Marine Dissolved Organic Matter Shares Thousands of Molecular Formulae Yet Differs Structurally across Major Water Masses. Environ. Sci. Technol. 2022, 56, 3758–3769. 10.1021/acs.est.1c04566. 35213127

[ref45] Lechtenfeld O. J.; Kattner G.; Flerus R.; McCallister S. L.; Schmitt-Kopplin P.; Koch B. P. Molecular Transformation and Degradation of Refractory Dissolved Organic Matter in the Atlantic and Southern Ocean. Geochim. Cosmochim. Acta 2014, 126, 321–337. 10.1016/j.gca.2013.11.009.

[ref46] Hertkorn N.; Frommberger M.; Witt M.; Koch B. P.; Schmitt-Kopplin Ph.; Perdue E. M. Natural Organic Matter and the Event Horizon of Mass Spectrometry. Anal. Chem. 2008, 80, 8908–8919. 10.1021/ac800464g. 19551926

[ref47] Petras D.; Koester I.; Da Silva R.; Stephens B. M.; Haas A. F.; Nelson C. E.; Kelly L. W.; Aluwihare L. I.; Dorrestein P. C. High-Resolution Liquid Chromatography Tandem Mass Spectrometry Enables Large Scale Molecular Characterization of Dissolved Organic Matter. Front. Mar. Sci. 2017, 4, 405 10.3389/fmars.2017.00405.

[ref48] Mitchell P. J.; Simpson A. J.; Soong R.; Simpson M. J. Nuclear Magnetic Resonance Analysis of Changes in Dissolved Organic Matter Composition with Successive Layering on Clay Mineral Surfaces. Soil Syst. 2018, 2, 8 10.3390/soils2010008.

[ref49] Lam B.; Baer A.; Alaee M.; Lefebvre B.; Moser A.; Williams A.; Simpson A. J. Major Structural Components in Freshwater Dissolved Organic Matter. Environ. Sci. Technol. 2007, 41, 8240–8247. 10.1021/es0713072. 18200846

[ref50] Morris K. F.; Cutak B. J.; Dixon A. M.; Larive C. K. Analysis of Diffusion Coefficient Distributions in Humic and Fulvic Acids by Means of Diffusion Ordered NMR Spectroscopy. Anal. Chem. 1999, 71, 5315–5321. 10.1021/ac9907585. 21662729

[ref51] Arakawa N.; Aluwihare L. I.; Simpson A. J.; Soong R.; Stephens B. M.; Lane-Coplen D. Carotenoids Are the Likely Precursor of a Significant Fraction of Marine Dissolved Organic Matter. Sci. Adv. 2017, 3, e1602976 10.1126/sciadv.1602976. 28959723 PMC5617377

[ref52] Baldock J. A.; Skjemstad J. O. Role of the Soil Matrix and Minerals in Protecting Natural Organic Materials against Biological Attack. Org. Geochem. 2000, 31, 697–710. 10.1016/S0146-6380(00)00049-8.

[ref53] Preston C. M.; Tony Trofymow J. A.; Niu J.; Sayer B. G. 13C Nuclear Magnetic Resonance Spectroscopy with Cross-Polarization and Magic-Angle Spinning Investigation of the Proximate-Analysis Fractions Used to Assess Litter Quality in Decomposition Studies. Can. J. Bot. 1997, 75, 1601–1613. 10.1139/b97-872.

[ref54] Salloum M. J.; Chefetz B.; Hatcher P. G. Phenanthrene Sorption by Aliphatic-Rich Natural Organic Matter. Environ. Sci. Technol. 2002, 36, 1953–1958. 10.1021/es015796w. 12026977

[ref55] Simpson M. J.; Otto A.; Feng X. Comparison of Solid-State Carbon-13 Nuclear Magnetic Resonance and Organic Matter Biomarkers for Assessing Soil Organic Matter Degradation. Soil Sci. Soc. Am. J. 2008, 72, 268–276. 10.2136/sssaj2007.0045.

[ref56] Malcolm R. L. Applications of Solid-State 13C NMR Spectroscopy to Geochemical Studies of Humic Substances Humic Substances II. In Search of Structure; Hayes M. H. B., MacCarthy P., Malcolm R. L., Swift S., Eds.; Wiley: New York, USA, 1989; pp 340–372.

[ref57] Dittmar T.; Stubbins A. 12.6—Dissolved Organic Matter in Aquatic Systems. In Treatise on Geochemistry; Holland H. D., Turekian K. K., Eds., 2nd ed.; Elsevier: Oxford, 2014; pp 125–156.

[ref58] Thorn K. A.; Folan D. W.; MacCarthy P. Characterization of the International Humic Substances Society Standard and Reference Fulvic and Humic Acids by Solution State Carbon-13 (13C) and Hydrogen-1 (1H) Nuclear Magnetic Resonance Spectrometry; Water-Resources Investigations Report 89–4196; U.S. Geological Survey: Denver, Colorado, 1989. https://pubs.usgs.gov/wri/1989/4196/report.pdf (accessed Oct 26, 2022).

