# Supplementary material for: Characterization of a Newly Available Coastal Marine Dissolved Organic Matter Reference Material (TRM-0522)
Source: Anal Chem. 2023 Apr 13;95(16):6559–67. doi: 10.1021/acs.analchem.2c05304 (PMC10134136; doi:10.1021/acs.analchem.2c05304)
Supplement: Supplementary file 1 — ac2c05304_si_001.pdf [file ac2c05304_si_001.pdf]

Characterisation of a newly available coastal marine dissolved organic matter reference material (TRM-0522)

Stacey L. Felgate[1], Alexander Craig[1,2], Lindon Moodie[2] and Jeffrey Hawkes[1*]

[1]Analytical Chemistry, Department of Chemistry BMC, Uppsala University, Uppsala 752 37, Sweden
[2] Drug Design and Discovery, Department of Medicinal Chemistry, Uppsala University, Uppsala 752 37, Sweden

*Corresponding author: J. Hawkes (jeffrey.hawkes@kemi.uu.se)

Contents

Text S1: Details of Tjärnö seawater system
Text S2: Details of column pre-conditioning
Text S3. Details of removal of residual column material

Figure S1. Map showing location of water extraction point.
Figure S2. Salinity and temperature data from the Tjärnö Marine Laboratory intake.
Figure S3: Absorbance scan and excitation – emission matrix for TRM-0522.

Table S1: Coble Peak locations, descriptions, and values for TRM-0522.
Table S2: Resolved peak metrics from LC-MS/MS

Text S1: Details of Tjärnö seawater system
Water used in this DOM extraction was drawn from a 45 m deep intake located ~1.0 km off-shore of the nearest land mass on the west coast of Sweden at 58°52.843 N 11°06.378 E (Figure S1). Water from this intake flows through a 10 mm coarse sieve before being pumped through a three-layer sand filtration system (6 - 8 mm; 3 - 4 mm; 1 - 2 mm) into a water reservoir. This reservoir feeds an extensive aquarium seawater system which includes a series of seawater taps (www.gu.se/en/tjarno/study-and-work/sea-water-systems). Water was drawn from this system on a daily basis between 16th and 20th May, 2022, during which time salinity (mean ± standard deviation = 33.3 ± 0.2 PSU; range = 32.9 - 33.7 PSU) and water temperature (mean ± standard deviation = 9.6 ± 0.5 °C ℃; range = 9.0 - 10.4 °C) at the intake were relatively stable (Figure S2).

Text S2: Details of column pre-conditioning
A PuriFlash C18-AQ F1600 (Interchim) column was pre-conditioned with ~ 5 column volumes (12.5 L) of LCMS grade methanol (MeOH), starting at a rate of 20 mL min^{-1} and gradually increasing to 250 mL min^{-1} (4.5 bar pressure), followed by ~ 10 column volumes (25 L) MilliQ water acidified to pH 2.0 using ACS grade hydrochloric acid (HCl), at a rate of 400 mL min^{-1} (4 bar pressure). An operating pH of 2 was initially used based on standard protocols (e.g. Dittmar et al. 2008) but this appeared to strip a small amount of material from the column, and so the operating pH was raised to 3. A further 2.5 L acidified MilliQ and 2.5 L 100 % MeOH were passed through the column at a reduced flow rate to 250 mL min^{-1} (4.5 bar pressure), and then it was stored in 50 % MeOH for 4 days. Immediately prior to use, the column was flushed with 5 L of 50 % MeOH, 5 L of 20 % MeOH, and 5 L acidified MQ (pH 3) with the flow rate gradually increased from 20 mL min^{-1} to 180 mL min^{-1} (3 - 4 bar pressure). On the first day the flow rate was gradually increased from 180 mL min^{-1} (3.5 bar pressure) to 330 mL min^{-1} (4.5 bar pressure) over a period of 3 hours. A flow rate of between 320 and 360 mL min^{-1} and 4 bar pressure was maintained for the rest of the extraction.

Text S3. Details of removal of residual column material
Each daily fraction was redissolved in 50 % MeOH and centrifuged for 15 mins at 6500 rpm. A white precipitate was pelleted out allowing the supernatant sample to be pipetted from the top. These samples were then freeze-dried and weighed. The total mass of these freeze-dried fractions was 1.8 g. Each fraction was redissolved in 50 % MeOH and combined to produce a single 115 mL sample. Triplicate 10 µl sub-samples were dried down in a Speedvac drying centrifuge and re-diluted in 11 mL MilliQ water for measurement of dissolved organic carbon (DOC). DOC concentrations in these samples were 4.74 ± 0.44 mg L^{-1} (mean ± standard deviation), or 5297 ± 48.6 mg L^{-1} in the concentrated sample. This was equivalent to just 34 % of the total sample mass, with DOC typically representing > 40 % DOM in aquatic samples. A further processing step was applied to ensure complete removal of any residual column material which might be adding to the total sample mass. The mixture of material was dissolved in 500 mL of a 0.1% solution of 25% ammonium hydroxide in LCMS grade water. This mixture was washed with 500 mL of $CHCl_3$ three times. This led to three separate liquid components: an aqueous phase, an organic phase, and an emulsion layer. The aqueous and organic phases were concentrated under reduced pressure using a rotary evaporator, then freeze dried. The emulsion layer (~ 100 mL) was mixed with 500 mL of a 0.1% solution of 25 % NH_4OH in LCMS grade water and stirred for 30 minutes to break

down the majority of the emulsion. The mixture was then washed with 500 mL of $CHCl_3$ another three times. Again, this led to an aqueous phase, an organic phase, and an emulsion layer. These liquid components were concentrated under reduced pressure, before being freeze dried. Five separate dried components were obtained: two aqueous, two organic, and one from the emulsion. ^{1}H NMR and LC-MS analysis of these five fractions showed that the material presumed to be from the column had been transferred to the organic phases, while the vast majority of the DOM had remained in the aqueous phases. While both the column material and the DOM were observed in the emulsion fraction, this accounted for just ~ 2% of the total dried mass and was discarded, along with the organic portions. The two aqueous portions were re-dissolved in 50 mL of a 0.1% solution of 25% NH_4OH in LCMS grade water, combined, dried under reduced pressure, and freeze dried to produce a dried mass of 1058 mg. This was re-dissolved in 100 mL of 10 % MeOH in MilliQ water with 0.1 % NH_3, aliquoted for distribution, and dried down a final time using a Speedvac (1 mg and 10 mg aliquots) or freeze drier (larger aliquots). A 1 mg sub-sample was re-diluted in 41 mL MilliQ water, giving a DOM concentration of 24.7 mg L^{-1}. DOC concentration in this sample was 10.1 mg L^{-1}, equivalent to 41 % DOM, which is in line with expectations and, along with detailed characterisation data (see later), suggests that removal of column material was successful.

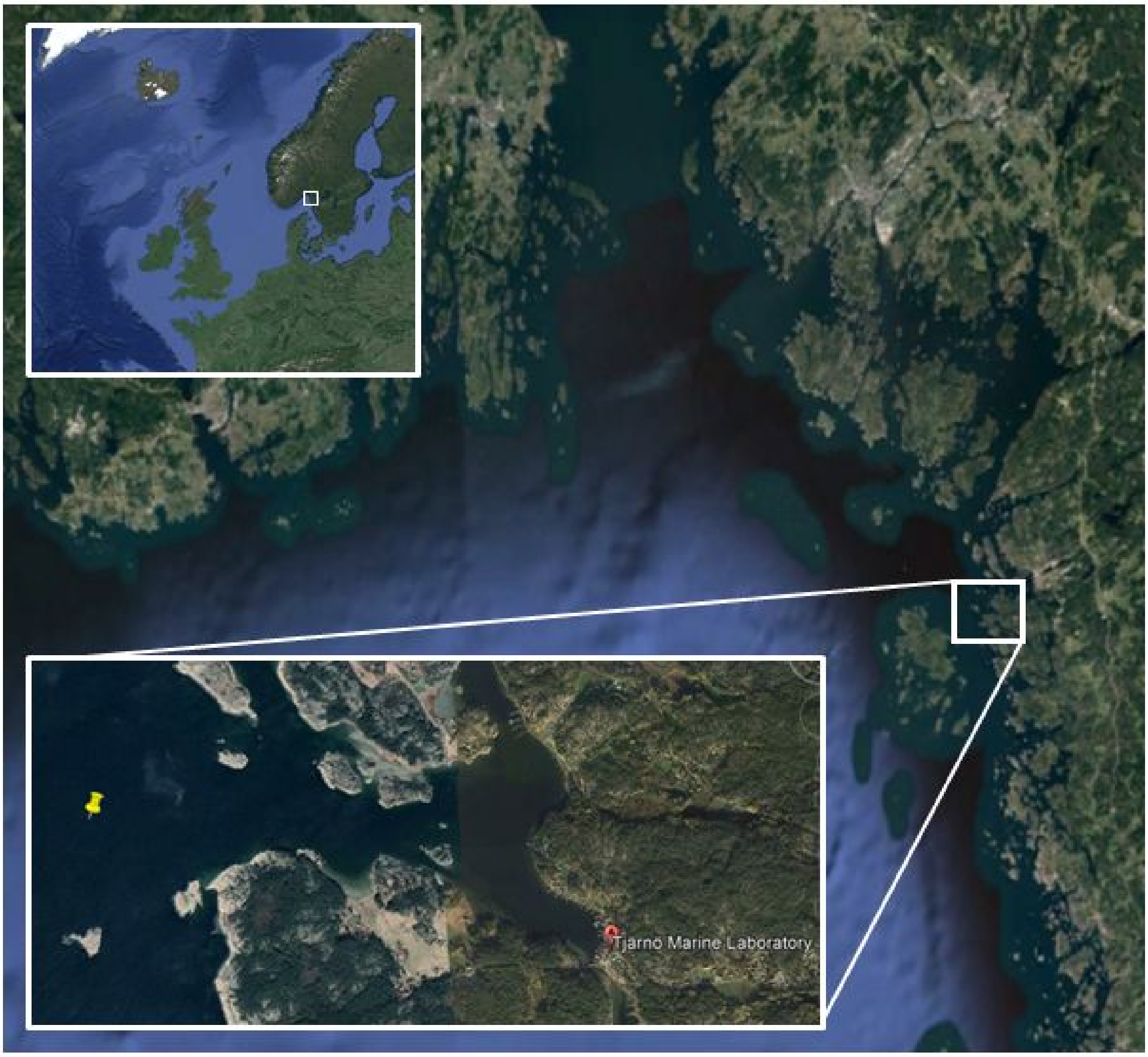

Figure S1. Map showing location of Tjärnö Marine Laboratory (red pin) and seawater intake (yellow pin) on the west coast of Sweden. Images provided by Landsat / Copernicus via Google Earth, and © TerraMetrics (main image) and CNES / Airbus (lower insert).

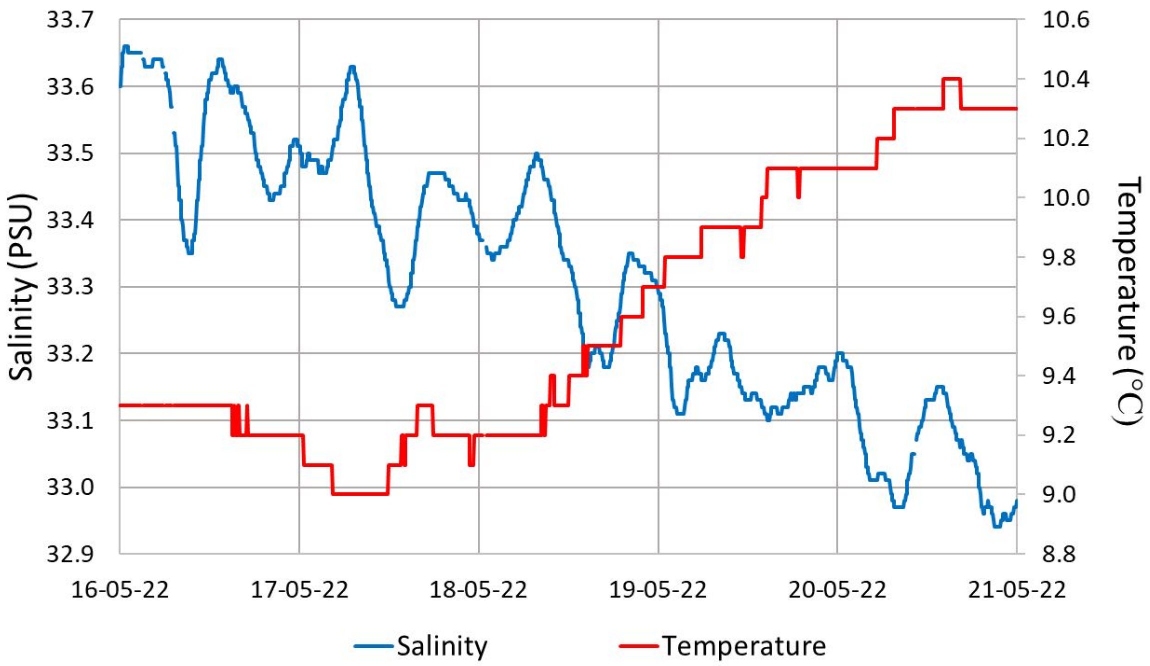

Figure S2. Five-minute resolution salinity (blue) and water temperature (red) data from the Tjärnö Marine Laboratory deep water intake during the extraction period (16th - 20th May, 2022). Data provided by Gothenburg University (www.weather.mi.gu.se/tjarno/data.shtml).

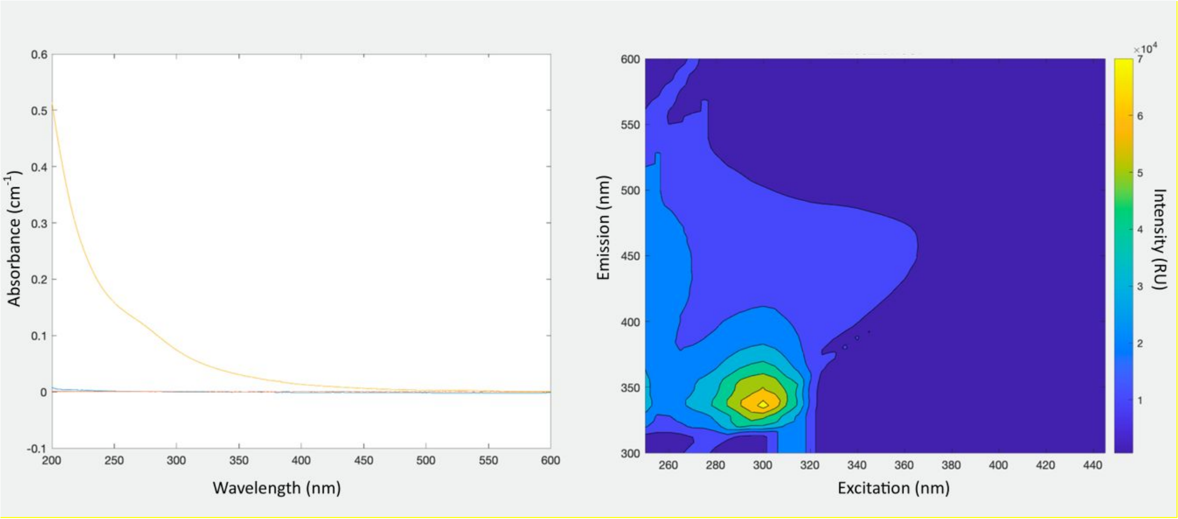

Figure S3. Absorbance scan (left; MilliQ absorbance blanks are shown in red and blue and TRM-0522 is shown in yellow) and excitation – emission matrix (right) for TRM-0522.

Table S1. Coble Peak locations, descriptions, and values for TRM-0522.

Coble Peak	Ex (nm)	Em (nm)	Description	Value
T	275	340	Tyrosine-like	3.91
M	312	380 - 420	Marine Humic-like	2.93
A	260	380 - 460	Terrestrial humic-like	2.71
B	275	310	Tryptophan-like	1.66
C	350	420 - 480	Humic-like	1.45

Table S2. Resolved peak metrics from LC-MS/MS (* From MZmine3, ¥ From GNPS, see data sharing).

Mode	MS1 features (with MS2 trigger)*	MS2 clusters¥	Library matches¥
Positive	261 (200)	130	5
Negative	233 (163)	299	5
